# Reduced myocardial perfusion is common among subjects with ischemia and no obstructive coronary artery disease and heart failure with preserved ejection fraction: a report from the WISE-CVD continuation study

**DOI:** 10.20517/2574-1209.2021.103

**Published:** 2022-03-05

**Authors:** Haider Aldiwani, Michael D. Nelson, Behzad Sharif, Janet Wei, T. Jake Samuel, Nissi Suppogu, Odayme Quesada, Galen Cook-Wiens, Edward Gill, Lidia S. Szczepaniak, Louise E. J. Thomson, Balaji Tamarappoo, Anum Asif, Chrisandra Shufelt, Daniel Berman, C. Noel Bairey Merz

**Affiliations:** 1 Barbra Streisand Women’s Heart Center, Cedars-Sinai Smidt Heart Institute, Los Angeles, CA 90048, USA.; 2 Applied Physiology and Advanced Imaging Laboratory, University of Texas, Arlington, TX 76019, USA.

**Keywords:** Non-obstructive coronary artery disease, cardiac magnetic resonance imaging, coronary microvascular dysfunction, heart failure with preserved ejection fraction

## Abstract

**Aim:**

Women with evidence of ischemia and no obstructive coronary artery disease (INOCA) have an increased risk of major adverse cardiac events, including heart failure with preserved ejection fraction (HFpEF). To investigate potential links between INOCA and HFpEF, we examined pathophysiological findings present in both INOCA and HFpEF.

**Methods:**

We performed adenosine stress cardiac magnetic resonance imaging (CMRI) in 56 participants, including 35 women with suspected INOCA, 13 women with HFpEF, and 8 reference control women. Myocardial perfusion imaging was performed at rest and with vasodilator stress with intravenous adenosine. Myocardial perfusion reserve index was quantified as the ratio of the upslope of increase in myocardial contrast at stress *vs*. rest. All CMRI measures were quantified using CVI42 software (Circle Cardiovascular Imaging Inc). Statistical analysis was performed using linear regression models, Fisher’s exact tests, ANOVA, or Kruskal-Wallis tests.

**Results:**

Age (*P* = 0.007), Body surface area (0.05) were higher in the HFpEF group. Left ventricular ejection fraction (*P* = 0.02) was lower among the INOCA and HFpEF groups than reference controls after age adjustment. In addition, there was a graded reduction in myocardial perfusion reserve index in HFpEF *vs*. INOCA *vs*. reference controls (1.5 ± 0.3, 1.8 ± 0.3, 1.9 ± 0.3, *P* = 0.02), which was attenuated with age-adjustment.

**Conclusion:**

Reduced myocardial perfusion reserve appears to be a common pathophysiologic feature in INOCA and HFpEF patients.

## INTRODUCTION

Women with evidence of ischemia and no obstructive coronary artery disease (INOCA) are at increased risk of developing major adverse cardiovascular events, most commonly heart failure with preserved ejection fraction (HFpEF)^[1–3]^. Up to 50% of women with INOCA have coronary microvascular dysfunction (CMD) with impaired myocardial perfusion reserve, most often detected by reduced coronary flow reserve through invasive coronary function testing (CFT) or non-invasive imaging such as cardiac magnetic resonance imaging (CMRI) or positron emission tomography^[4,5]^. Previous studies demonstrated that women with CMD often have left ventricular (LV) diastolic dysfunction, adverse ventricular remodeling, elevated inflammatory markers, and myocardial scar^[6–8]^. However, the mechanism(s) linking HFpEF with INOCA remains to be elucidated. Accordingly, we hypothesized that CMD might be contributing to adverse ventricular remodeling in INOCA and subsequently to HFpEF. We tested our hypothesis by evaluating LV remodeling and myocardial perfusion abnormalities in participants with CMD, HFpEF, and reference controls.

## METHODS

**Figure 1.**
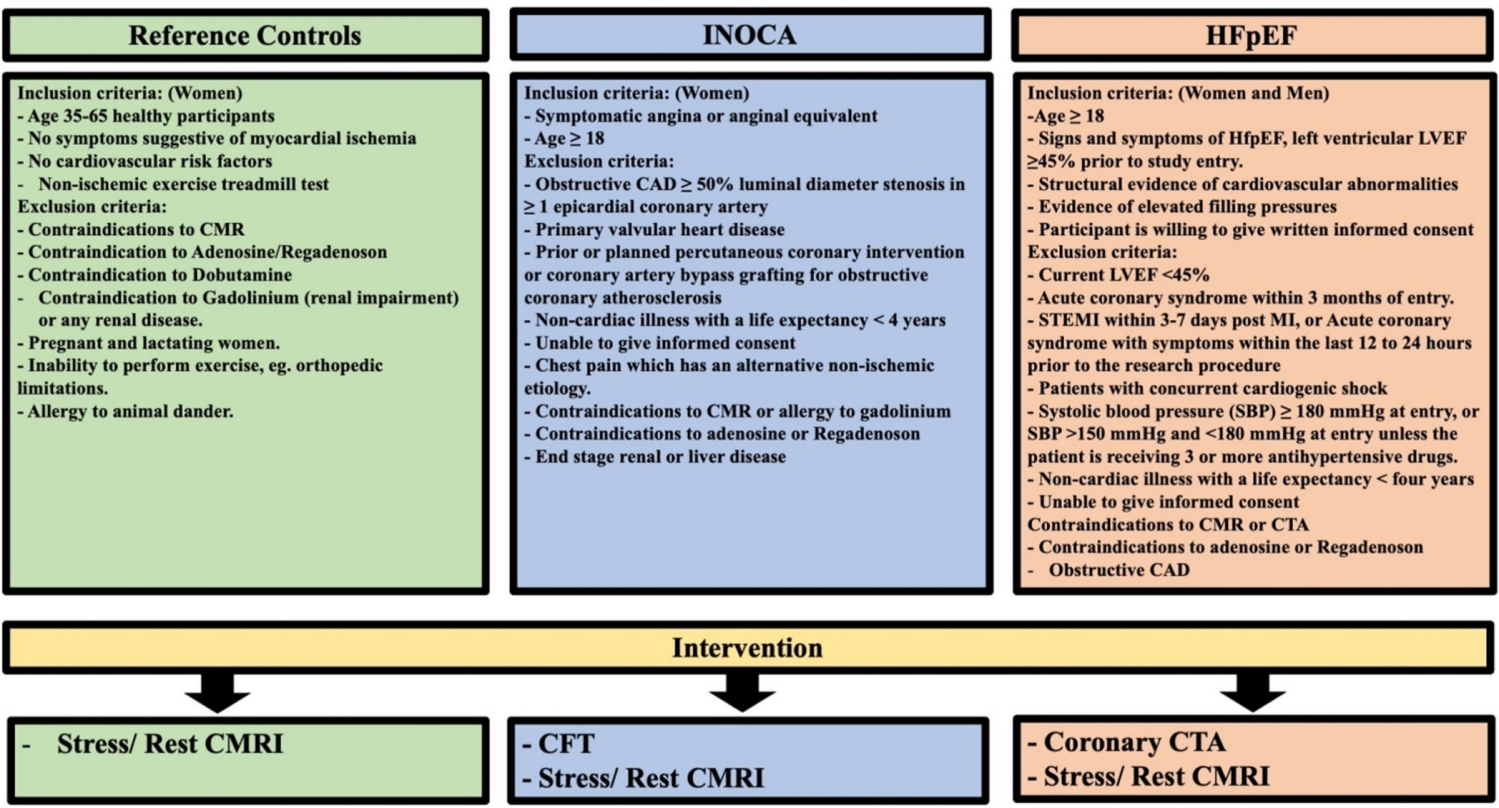
Illustrating study participants selection criteria and the selected intervention for each group. CMRI: Cardiac magnetic resonance imaging; INOCA: ischemia with no obstructive coronary artery; CAD: coronary artery disease; HFpEF: heart failure with preserved ejection fraction; LVEF: left ventricular ejection fraction; STEMI: ST elevation myocardial infarction; MI: myocardial infarction; CTA: computed tomography angiography.

Sixty-four participants were recruited, including 36 women with suspected INOCA, 20 with HFpEF (14 women and 6 men), and 8 reference control women. All study participants selection criteria and intervention are illustrated in Figure 1. Participants in the INOCA and the HFpEF groups were recruited from the Women’s Ischemia Syndrome Evaluation - Coronary Vascular Dysfunction (WISE-CVD) Continuation Study, also known as Women’s Ischemia Syndrome Evaluation - CMD and HFpEF at Cedars-Sinai Medical Center, Los Angeles, CA NCT02582021. All women with INOCA underwent clinically indicated invasive CFT after demonstration of no obstructive coronary artery disease (CAD), defined as < 50% stenosis. CFT is considered the gold standard invasive test to diagnose CMD and published previously^[9]^. In brief, CFT was performed by infusing vasoactive substances through a guiding catheter placed in the left main coronary artery. The Doppler guide wire was positioned in the proximal left anterior descending coronary artery, and test results are interpreted in Table 1^[9]^.

All participants hospitalized for HFpEF were enrolled. All participants met modified criteria for diagnosis of HFpEF based on the European Society of Cardiology guidelines (2012)^[10]^, including symptoms of heart failure (e.g., dyspnea, orthopnea, paroxysmal nocturnal dyspnea, and edema), left ventricular ejection fraction > 45% prior to study entry, structural evidence of cardiovascular abnormalities: evidence of abnormal filling or relaxation, left atrial enlargement or left ventricular hypertrophy documented by echocardiogram and evidence of elevated filling pressure pressures: left ventricular end-diastolic pressure or pulmonary capillary wedge pressure at rest > 15 mmHg, elevated brain natriuretic peptide, or use of diuretics. In addition, participants who had the following were excluded: LVEF < 45%, Acute coronary syndrome (defined by ACC/AHA guidelines, including MI^[10]^ within 3 months of entry, patients who have had an MI or other event within the 6 months prior to entry unless an echocardiogram measurement performed after the event confirms a LVEF ≥ 45%, primary valvular heart disease (> moderate regurgitation or > mild stenosis), primary cardiomyopathies (hypertrophic, infiltrative or restrictive), constrictive pericarditis, high-output heart failure and right ventricular myopathies, patients with concurrent cardiogenic shock or requiring inotropic or intra-aortic balloon support or current acute decompensated HF requiring therapy, alternative reason for dyspnea such as: significant pulmonary disease or severe COPD, hemoglobin less than 10 g/dL or body mass index more than 40 kg/m^2^, systolic blood pressure (SBP) ≥ 180 mmHg at entry, or SBP > 150 mmHg and < 180 mmHg at entry unless the patient is receiving 3 or more antihypertensive drugs, prior or planned percutaneous coronary intervention or coronary artery bypass grafting, Non-cardiac illness with a life expectancy less than 4 years, inability to give informed consent, contraindication to CMRI, end stage renal disease, end stage liver disease. In addition, obstructive CAD was excluded by noninvasive coronary computed tomography angiography in this group due to the lack of clinically indicated CFT. Reference control women were recruited from the Cardiac Magnetic Resonance Imaging Normal Reference Control Group Testing study NCT00573339.

Women had no symptoms suggestive of cardiovascular ischemia and cardiovascular risk factors and had a non-ischemic Bruce protocol exercise treadmill stress test. The study was approved by the institutional review board, and all participants provided written informed consent.

For stress, all participants received either adenosine (*n* = 59) (140 μg/kg for 3 min) or regadenoson (*n* = 5) stress-rest perfusion (0.4 mg). CMRI was per) and a standardized gadolinium bolus of 0.05 mmoL/kg injected at 4 mformed using standardized product sequences (3T Siemens Healthcare, Erlangen, GermanyL/s (Gadavist gadobutrol injection 1 mmoL/mL) during stress and rest. First-pass perfusion images were obtained in three LV short-axis slices (basal, mid, and distal LV slice positions) with the following parameters: Gradient echo - EPI hybrid sequence, TR per slice 134.8 ms, TE 0.94 ms, BW 1240 Hz/pixel, readout flip angle 43°, slice thickness 8 mm, image matrix 155 × 224 pixels, in-plane resolution 1.34 mm × 1.34 mm × 8 mm^2^, parallel imaging (GRAPPA) factor 2, imaging three slices every heartbeat. In the event of a peak stress heart rate of > 120 bpm, two slices were obtained during stress first-pass imaging with the exclusion of the distal LV slice position. Cardiac morphology and function were assessed using a stack of short-axis cine images spanning the entire LV, together with a series of long-axis images in the horizontal, vertical, and LV outflow tract views. Typical scan parameters included contiguous 8 mm/0 mm slices, 1.34 mm × 1.34 mm × 8 mm voxel size, 155 mm × 224 mm matrix, 25 cardiac phases, 11 segments, 10 heartbeats/slice. Visual assessment of splenic switch-off for evaluation of stress test adequacy was performed in participants who received adenosine.

All CMRI data were quantified and analyzed using CVI42 software (Circle Cardiovascular Imaging Inc.). Myocardial perfusion reserve index (MPRI) was calculated as previously published^[5]^. The Seattle Angina Questionnaire (SAQ), measuring five functional dimensions, was used to characterize clinical symptoms (higher scores indicate less angina). Kansas City Cardiomyopathy Questionnaire (KCCQ) was used to characterize heart failure symptoms (higher scores indicate better HF symptoms). Left ventricular mass and end-systolic and end-diastolic volumes were indexed to the body surface area.

**Figure 2.**
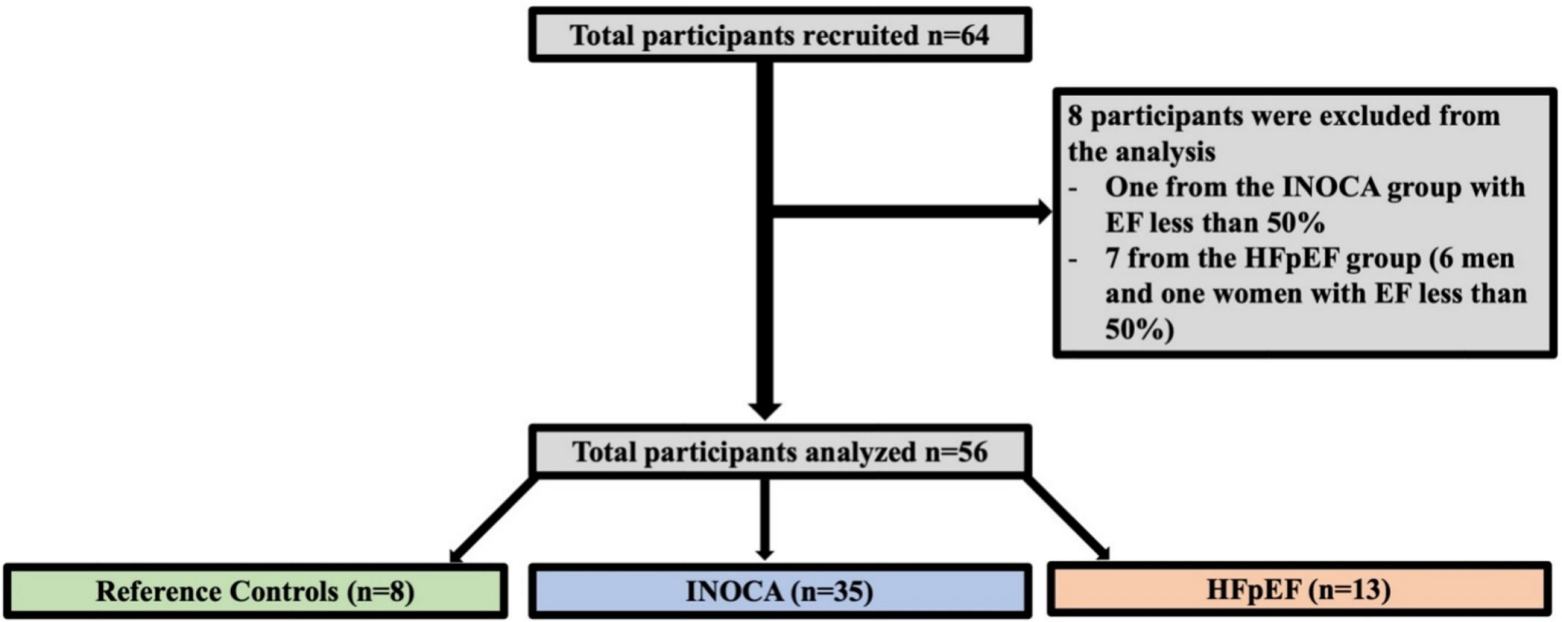
Illustrating the total study participant enrolled and total study participants analyzed. INOCA: Ischemia with no obstructive coronary artery; HFpEF: heart failure with preserved ejection fraction; EF: ejection fraction.

We analyzed a total of 56 women participants and excluded men participants and participants with an EF of less than 50%, as shown in Figure 2 according to the most recent ESC guidelines to diagnose HFpEF^[11]^.

### Statistical analysis

Age-adjusted p-values were statistically obtained using linear regression models, and non-adjusted *P*-values were obtained using either Fisher’s exact tests, ANOVA, or Kruskal-Wallis tests using SAS software.

## RESULTS

Pertinent baseline characteristics are summarized in Table 1. Age and body surface area (BSA) differed among the groups; participants with HFpEF were the oldest and had the highest BSA compared to women with INOCA and reference controls. Participants with INOCA and HFpEF shared different percentages of common cardiovascular risk factors, including hypertension, diabetes mellitus (DM), dyslipidemia, former smoking history, and family history of premature CAD. Essential hypertension and DM were prevalent among the HFpEF group compared to the INOCA group (75%, 40%, *P* = 0.003, 30.8%, 6.5%, *P* = 0.05) while family history of CAD was prevalent among the INOCA group compared to the HFpEF group (71%, 46.2%, *P* = 0.04). Both groups had similar use of calcium channel blockers, aspirin, angiotensin-converting enzyme inhibitors, angiotensin receptor blockers, and statins. The β-blocker use was more prevalent in the HFpEF group than in the INOCA (80%, 22.2%, *P* = 0.002). As expected, the INOCA group had lower SAQ scores compared to the HFpEF group, indicating relatively severe angina symptoms. Interestingly, KCCQ symptoms were modestly reduced among the INOCA and the HFpEF groups, and there were no statistically significant differences between them across all the domains.

CMRI results are shown in Table 1. LV ejection fraction differed across the groups after adjusting for age and demonstrating a significant decrease among HFpEF and INOCA groups compared to reference controls. LV volumes, LV mass, LV mass to volume ratio were not statistically different across the groups. Consistent with our hypothesis, we observed a graded reduction in subendocardial, subepicardial, and transmural MPRI among HFpEF, INOCA, and reference controls. These findings were attenuated by age adjustment; however, epicardial MPRI remained statistically significant across the groups. Results for the CFT among the INOCA population are illustrated in Table 1.

## DISCUSSION

The data supports the hypothesis that reduced myocardial perfusion is a common pathophysiologic feature in INOCA and HFpEF patients. These data show a reduction in LVEF and impaired myocardial perfusion reserve in patients with suspected INOCA and patients with HFpEF. The perfusion abnormality was worse among participants with HFpEF than INOCA and raised the question of whether INOCA and HFpEF share similar pathophysiological processes but worse in HFpEF than INOCA. Although myocardial perfusion was reduced in both INOCA and HFpEF, only the epicardial MPRI remained statistically significantly different following age adjustment. Similar to women with HFpEF, women with INOCA showed significant symptoms related to HF evidenced by the KCCQ assessment.

Women with suspected INOCA are at increased risk of developing major adverse cardiovascular events, most commonly HFpEF^[1–3]^; however, the contributing mechanism(s) to subsequent heart failure progression in INOCA has not been well characterized. Recent results from the PROMIS-HFpEF trial showed a high prevalence of CMD evident from reduced coronary flow reserve measured using adenosine stress transthoracic Doppler echocardiography among HFpEF patients^[12]^. Furthermore, emerging data support the hypothesis that myocardial ischemia, secondary to CMD, is a key mechanism leading to pathological remodeling in HFpEF^[4,13,14]^. Indeed, the impaired coronary microvascular function has been independently associated with features of HFpEF such as LV diastolic dysfunction and elevated highsensitivity cardiac troponin concentrations, leading to worse clinical outcomes and recurrent hospitalizations^[4,13]^. Like CMD, other cardiovascular risk factors such as essential hypertension and DM can adversely impact cardiac remodeling^[15]^. Previous studies demonstrated that CMD and HFpEF share similar risk factors, including age, obesity, hypertension, and DM^[5,16–19]^. The current investigation builds upon these prior observations by directly comparing indices of LV morphology and myocardial perfusion in INOCA and HFpEF as compared to reference control participants.

A possible explanation for our findings is that participants in the HFpEF group are older, more obese, have a higher prevalence of DM, relatively more hypertensive, and therefore at greater risk of chronic myocardial microvascular ischemia evidenced by reduced perfusion. Thus, it is plausible that chronic exposure to myocardial ischemia may be linked to adverse remodeling.

Our study is limited by relatively small sample size and, therefore, limited in adjusting for confounding variables. Our study is also limited by the lack of a longitudinal design and the inability to decide the temporality of our findings. In addition, our focus on the INOCA and reference control groups of exclusive women limits the generalizability of these results to men. Finally, our recruitment of symptomatic INOCA participants limits generalizability to asymptomatic patients.

These data, taken together, support the hypothesis that CMD and other cardiovascular risk factors may play a pivotal role in driving heart failure progression in INOCA. However, future studies are needed to confirm the exact mechanistic link through longitudinal investigation of women with INOCA.

## Figures and Tables

**Table 1.** Baseline and age-adjusted characteristics and CMRI variables between reference controls, INOCA, and HFpEF

Variable	Reference control (*n* = 8)	INOCA (*n* = 35)	HFpEF (*n* = 13)	*P*-value	Age-adjusted *P*-value

**Baseline characteristics**					
Age	48 (5)	55 (10)	62 (12)	**0.007**	-
BSA (m^2^)	1.7 (0.1)	1.7 (0.2)	1.8 (0.2)	**0.05**	0.08
**Risk factors**					
Hypertension	0	12 (40.0%)	9 (75.0%)	**0.003**	-
Diabetes mellitus	0	2 (6.5%)	4 (30.8%)	**0.05**	-
Former smoker	0	10 (32.3%)	6 (46.2%)	0.5	-
Current smoker	0	2 (6.5%)	1 (7.7%)	1	-
Dyslipidemia	1 (12.5%)	2 (8.3%)	1 (10.0%)	1	-
Family history of CAD	2 (25%)	22 (71%)	6 (46.2%)	**0.04**	-
**Medications**					
Beta blockers	0	6 (22.2%)	8 (80%)	**0.002**	-
Calcium channel blockers	0	13 (48.1%)	4 (50%)	1	-
Nitrates	0	12 (48%)	2 (20%)	0.25	-
Aspirin	0	23 (82.1%)	7 (63.6%)	0.24	-
ACEIs	0	10 (35.7%)	2 (20%)	0.45	-
ARBs	0 (0.0%)	1 (3.8%)	0	1	-
Statins	0	19 (67.9%)	6 (54.5%)	0.48	-
SAQ-7 questionnaire	-	52.1 (19.5)	70.2 (30)	**0.02**	0.09
KCCQ clinical summary	-	70.1 (18.8)	62.9 (22.3)	0.28	0.11
KCCQ overall summary	-	62.7 (22.7)	62.6 (24.1)	0.99	0.49
KCCQ physical limitation	-	73.6 (19.9)	68.5 (27.9)	0.5	0.27
KCCQ quality of life	-	48.9 (29.4)	59.0 (33.1)	0.33	0.7
KCCQ symptom burden	-	63.6 (22.9)	58.3 (27)	0.51	0.25
KCCQ self-efficacy	-	67.9 (29.8)	80.8 (18.1)	0.16	0.26
KCCQ symptom frequency	-	69.4 (20.8)	60.9 (24.6)	0.25	0.07
KCCQ social limitation	-	61.6 (29.9)	66.0 (31.9)	0.68	0.85
KCCQ symptom stability	-	50.8 (24.1)	44.2 (11)	0.35	0.29
KCCQ total symptom score	-	66.5 (21.1)	59.6 (23)	0.34	0.12
**CMRI variables**					
LV EF (%)	66.6 (4.3)	61.8 (5.5)	62.0 (5)	0.07	**0.02**
LVEDV (mL)	105.7 (10.6)	115.7 (16.7)	113.0 (26.8)	0.4	0.35
LVEDV index (mL/m^2^)	63.5 (7.0)	68.5 (8.5)	62.9 (16.8)	0.21	0.24
LVESV (mL)	35.4 (6.1)	44.6 (11.2)	43.7 (15.1)	0.14	0.07
LVESV index (mL/m^2^)	21.3 (4.0)	26.3 (5.7)	24.3 (8.7)	0.12	0.07
LV stroke (mL)	70.3 (7.9)	71.1 (9.7)	69.3 (13.6)	0.87	0.84
LV mass (g)	67.8 (7.2)	76 (10.8)	81.9 (23.1)	0.1	0.31
LV mass to volume	0.6 (0.1)	0.7 (0.1)	0.7 (0.2)	0.09	0.4
LV mass index (g/m^2^)	40.6 (3.7)	44.9 (4.9)	45.1 (12)	0.28	0.47
**MPRI**					
MPRI transmural	1.9 (0.3)	1.8 (0.3)	1.5 (0.3)	**0.03**	0.13
MPRI epicardial	2.0 (0.3)	1.9 (0.3)	1.6 (0.3)	**0.01**	**0.03**
MPRI subendocardial	1.7 (0.3)	1.6 (0.3)	1.4 (0.3)	0.11	0.34
**CFT variables**					
CFR		2.9 ± 0.8			
Change in coronary artery diameter (%)		−1 ± 15.9			
Nitroglycerin response (%)		22.3 ± 32.7			
CBF (%)		48.3 ± 56			

BSA: Body surface area; MPRI: myocardial perfusion reserve index; SAQ: Seattle angina questionnaire; LVESV: left ventricular end-systolic volume; LVEDV: left ventricular end-diastolic volume; CFT: coronary function test; CFR: coronary flow reserve.

## Data Availability

Data available upon request.
